# Efficacy of Two Rotary Systems in Removing Gutta-Percha and Sealer from the Root Canal Walls

**Published:** 2011-05-15

**Authors:** Bahareh Dadresanfar, Payman Mehrvarzfar, Mohammad Ali Saghiri, Sedigh Ghafari, Zohreh Khalilak, Mehdi Vatanpour

**Affiliations:** 1 *Department of Endodontics, Dental School, Islamic Azad University of Medical sciences, Tehran, Iran.*; 2 *Department of Dental Materials, Dental School, Islamic Azad University of Medical sciences, Tehran, Iran.*; 3 *Dentist. Private practice, Tehran, Iran*

**Keywords:** Gutta-Percha, Mtwo R, Protaper D Series, Retreatment, SEM

## Abstract

**INTRODUCTION:** The aim of this *in vitro* study was to compare the efficacy of two retreatment rotary systems in removal of gutta-percha (GP) and sealer from the root canal walls with and without use of solvent.

**MATERIALS AND METHODS:** Sixty single-canalled distal roots of mandibular molars were prepared and root filled with gutta-percha and AH26. Each canal was randomly allocated to receive one of the retreatment techniques, Mtwo R or ProTaper. The groups were further divided into two subgroups: with or without the use of solvent. The cleanliness of canal walls was determined by stereomicroscope and scanning electron microscopy.

**RESULTS:** The results showed that Mtwo R without the use of solvent was more efficient in material removal compared to ProTaper D (P<0.05). Most remnants were found in the apical third of the canals (P<0.05).

**CONCLUSION:** Mtwo R seems to be an efficient rotary system for endodontic retreatment of root canal with GP.

## INTRODUCTION

Success of root canal retreatment depends on complete removal of the previous filling materials. Complete re-cleaning and re-shaping followed by proper filling of the root canal system is the key to reestablish healthy periradicular tissue (1-3).

Gutta-percha is one of the most popular root filling materials, and in cases of endodontic failure, various methods have been introduced to remove it from root canal system. These include rotary files, ultrasonic instruments, hand files combined with heat or chemicals, and paper point with chemicals (4,5). Currently, rotary instruments are becoming popular and most dentists prefer them to time consuming hand instrumentation techniques.

Two specific rotary systems have been designed by manufacturers for retreatment purposes including Mtwo R rotary files (Sweden & Martina, Padova, Italy) and the ProTaper Universal retreatment files (Dentsply, Maillefer, Ballaigues, Switzerland). Solvents such as chloroform allow quicker access to the working length and facilitate keeping to the original canal route (6). Their application along with specific retreatment rotary systems might improve canal cleanliness. It has been reported that both these systems left remnants of filling materials on canal walls although the effect of chemical solvent was not examined (7).

This *ex-vivo* study was conducted to compare the efficacy of two rotary retreatment systems on gutta-percha removal with and without solvent on extracted human teeth.

## MATERIALS AND METHODS

***Specimen Selection***

Sixty mandibular first molars were decoronated by means of diamond disc (D and Z, Berlin, Germany). A size 10 K-type file (Dentsply Maillefer, Ballaigues, Switzerland) was placed into the distal canal until it was visible at the apical foramen and the working length was established by reducing 1mm from this length. Buccolingual and mesiodistal radiographs (Ultraspeed Radiographic Film, Kodak, Rochester, MN, USA) were taken. Single-canalled distal roots with completely developed apices, and with angle of curvatures <20^º^ (8), similar root length (approximately 16mm) and apical root canal diameters no greater than size 20 K-file were selected.

***Canal Preparation and Obturation***

The root canals were prepared using step-back technique with K-files. The canals were enlarged to a #35 file as the master apical file (MAF) and flared to #60 file by reducing 0.5mm for each successive instrument.

During this procedure, each canal was irrigated with 2.5mL of 5.25% NaOCl after each instrument. In order to remove the smear layer, a final flush was performed with 5mL of 17% EDTA for 30 seconds followed by 5mL of 5.25% NaOCl for 30 seconds and then 5mL of distilled water. The canals were dried by proper paper points and obturated using lateral compaction method with the #35 gutta-percha point (Gapadent, Korea) as master gutta-percha cone and #15 cones as accessories AH26 (Dentsply, DeTrey, Konstanz, Germany) was utilized as sealer. In order to verify the root filling quality mesiodistal and buccolingual radiographs were taken. The orifice was sealed temporarily with Coltosol (Coltene, Altstatten, Switzerland) and the roots were incubated for 3 weeks at 37^º^ and 100% humidity.

***Retreatment Procedure***

The specimens were randomly divided into four groups with 15 roots each (n=15). The first 2-3mm of gutta-percha was removed with Gates-Glidden bur #2 (Dentsply Maillefer, Ballaigues, Switzerland) from the cervical part of the root. The retreatment instruments were carried into the canal using electric endo motor (Endo IT VDW, Munich, Germany). Speed and torque were set for each instrument according to the manufacturer’s instructions. Specimens in each group were retreated as follows:

***Group A*** *:* ***Mtwo R***

Mtwo R sizes 05/25 and 05/15 were used in a crown-down manner. Instrument size 05/15 was carried to the working length. Mtwo sizes 04/35 and 04/40 were used for final apical enlargement. The canals were rinsed with 5mL 5.25% NaOCl between each instrument.

***Group B*** *:* ***Mtwo R+solvent***

The specimens in this group were retreated in a same manner as group A except for receiving 2-3 drops of chloroform (Kimia Co. Tehran, Iran) before insertion of each Mtwo R instrument.

***Group C: ProTaper Universal retreatment files***

ProTaper Universal retreatment files (D1, D2, and D3) were used in a crown-down technique. Size D3 was used to working length. Final apical enlargement was performed by means of ProTaper size F4 (05/40).

***Group D*** *:* ***ProTaper Universal retreatment files+solvent***

The retreatment procedure was the same as group C, except 2-3 drops of chloroform were placed into the canal before instrumentation.

In all groups the canals were rinsed with 5mL of 5.25% NaOCl between each instrument. Retreatment was considered accomplished when gutta-percha was fully removed from the canal and none could be observed on the retreatment instruments. Subsequently, specimens were radiographed from mesiodistal and buccolingual directions to assure full debridement of the canal wall. All the procedures (root canal treatment and retreatment) were conducted by a trained operator.

***Evaluation***

The roots were split longitudinally by means of a diamond disk and chisel. Care was taken not to enter the canal lumen with disk. The amount of remaining gutta-percha and sealer was evaluated in three segments: 1mm above the apex (apical), 8mm from the apex (middle) and 2mm below the CEJ (coronal). Images were taken from all specimens in 3 segments by a digital camera attached to the stereomicroscope (OLYMPUS, SZM9) with ×16 magnifications.

**Figure 1:**
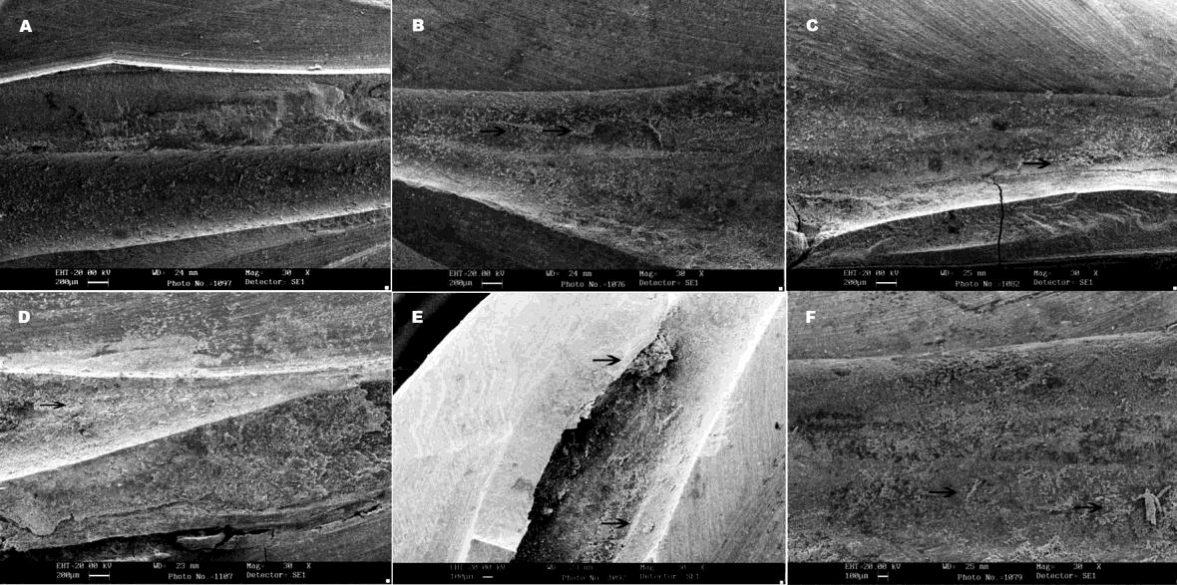
SEM images of filling remnants on root canal walls (×30): A) Mtwo R, B) Mtwo+solvent, C) ProTaper Universal retreatment, D) ProTaper Universal retreatment+solvent, E) More remnant material (→), and F) abnormal distribution of remnant GP are obvious in group C

In each specimen, the half which contained the greatest amount of filling material was selected. The ratio between dentinal wall and remnant material in each third of the canal was measured with the AutoCAD 2007 (Autodesk Inc., San Rafael, CA, USA).The images that showed no remaining material on canal walls were subjected to scanning electron microscopy SEM (Leo. 440i; Oxford Microscopy, Oxford, UK) using secondary electron detector (SE) analysis under ×30 magnification.

***Statistical Analysis***

The mean percentage of remnant gutta-percha, sealer and the comparison between groups was carried out using repeated measure ANOVA, Turkey’s Post Hoc test and Independent Sample t-test. Statistical significance was defined as P<0.05.

## RESULTS

The mean percentage and standard deviation of gutta-percha remnants on the canal wall surface for each studied group are presented in table 1. The Independent Sample t-test showed that Mtwo R left less filling remnants in all locations of the canal compared to ProTaper Universal, however the solvent adversely effected gutta-percha removal in coronal and middle thirds by Mtwo R (P<0.05).

Repeated measured ANOVA showed that there were significant differences between various areas of the samples in each group (P<0.05). There were no interactions between the two assessed factors (solvent and technique). Turkey’s Post Hoc test revealed that Mtwo R left significantly more remnants in apical third (P<0.05), and that ProTaper Universal left the most filling material in coronal third (P<0.05).

***Electron microscope***

All the samples selected from the 4 groups for SEM analysis revealed filling remnants, except one sample from Mtwo R group which showed no filling material on canal walls (Figure 1).

## DISCUSSION

Rotary instruments have been found very useful instruments in root canal retreatment (9-11). Schirrmeister *et al.* showed that RaCe rotary instruments were more efficient than FlexMaster and Hedström files for gutta-percha removal; however, they found that ProTaper rotary instrument was not significantly different to FlexMaster, Hedström files (10). They suggested that the greater ability of RaCe instruments in retreatment is due to the smooth surface of the instrument caused by the special chemical surface treatment and the resultant decrease in gutta-percha adherence to the flutes which increased the cutting ability. In the present study Mtwo R instruments showed better capability in gutta-percha removal than ProTaper Universal retreatment files. As mentioned in a study by Schäfer and Oitzinger Mtwo has a small core diameter, great chip removal capacity and great chip space that can result in great cutting ability (12). Accordingly, it seems logical to attribute Mtwo R performance in the present study due to its great cutting ability and surface treatment in which provides greater wet ability and cleaner canal walls. Finding more remnants in the apical third is in complete accordance with Somma *et al.*’s findings (6), suggesting the greater size of apical preparation when using Mtwo rotary instruments. The more gutta-percha remnants on coronal third with ProTaper Universal instruments could be attributed to their high centering ability and the wide shape of distal canal roots which were used in this study. Masiero and Barletta found more gutta-percha remnants in cervical third of root canals when using K3 instruments. They declared that the file had remained centered and had not touched all the walls in the wide cervical area (9). ProTaper Universal contains three flexible instruments (D1, D2 and D3), of which the tapers and diameters are 0.09/0.30mm, 0.08/0.25mm, and 0.07/0.20mm, respectively. Another study suggested that convex triangular cross section of D series instruments reduces their contact area with canal walls (13); this might be another reason for more filling remnants found in ProTaper group in the present study.

**Table 1:** Mean percentage and standard deviation of the remaining gutta-percha on the canal wall surface evaluated by means of stereomicroscope

**Group**	**Coronal**	**Middle**	**Apical**
**Mtwo R**	32.23±18.89	27.88±16.15	44.93±25.05
**Mtwo R with solvent**	48.37±24.77	46.09±18.31	40.82±17.56
**ProTaper**	71.63±15.89	58.41±20.24	58.69±18.43
**ProTaper with solvent**	70.61±16.76	57.87±20.83	59.58±20.57

In a recent study by Horvath *et al*. solvent application resulted in more gutta-percha remnants on canal walls and dentinal tubules (14). The application of solvent had adverse effect on Mtwo R group in the present study as well. It has been reported that Mtwo instruments with positive rake angles (15) act more like Hedström files and tend to remove bulks of filling material. Horvath study also showed that the solvent softens gutta-percha and the resultant chloropercha finds a viscose consistency. The authors assumed that this is responsible for the reduced retreatment ability of Mtwo R instruments in presence of solvent.

Chloroform has been used as a solvent in gutta-percha retreatment and its efficacy and benefits have been demonstrated in previous studies (7,16,17). This material has been introduced as the most efficient solvent in dissolving gutta-percha (18). Chloroform possesses antibacterial activity (19); on the other hand the International Agency for Research of Cancer has classified this solvent as group 2B of carcinogens which indicates inadequate evidence of carcinogenicity in humans, but sufficient evidence of carcinogenicity in experimental animals (10). According to the results of this study, we do not suggest using chloroform in accompany with Mtwo R instruments. Subsequently, antibacterial property can be achieved by the flushing agents and hand/rotary instruments designed for this purpose.

Since mesiodistally flattened anatomy is an important variable in retreatment cases (11), distal roots of mandibular molars were selected in this study. Decoronation of teeth assures standardization of specimens as it eliminates the effect of crown anatomy and the root canals’ access and increase the reliability of (16). Sample size in this study was in accordance with previous researches (5,6). The evaluating method for filling remnants can be indicative. Because of two-dimension presentation of radiographs, (1,5) longitudinal cleavage of roots was performed in this study to observe the remnants. According to previous studies (1,9), software was used in order to measure the remnants of sealer and gutta-percha on dentinal walls.

Although some specimens did not show filling remnants in radiographic examination and under stereomicroscope, but SEM analysis revealed filling materials in all samples except one. The challenge in retreatment cases is that the clinician can only resort to the visual and radiographic analysis for evaluating the cleansing thoroughness of canals following retreatment procedures (7).

## CONCLUSION

Under the conditions of the present *ex vivo* study, Mtwo R files are significantly more effective in removing gutta-percha from root canal walls compared to ProTaper Universal files; also chloroform as a solvent adversely affects the efficiencies of Mtwo R instruments.
